# Immunomodulation in Sepsis: The Role of Endotoxin Removal by Polymyxin B-Immobilized Cartridge

**DOI:** 10.1155/2013/507539

**Published:** 2013-10-22

**Authors:** Elisabeth Esteban, Ricard Ferrer, Laia Alsina, Antonio Artigas

**Affiliations:** ^1^Pediatric Intensive Care Unit, Hospital Sant Joan de Déu, Passeig Sant Joan de Déu, 2.08950, Universitat de Barcelona, Esplugues de Llobregat, Barcelona, Spain; ^2^Intensive Care Department, Mutua de Terrassa University Hospital, Pl. Dr. Robert, 5.08221, CIBER Enfermedades Respiratorias, Terrassa, Spain; ^3^Allergy and Clinical Immunology Department, Hospital Sant Joan de Déu, Passeig Sant Joan de Déu, 2.08950, Esplugues de Llobregat, Universitat de Barcelona, Barcelona, Spain; ^4^Critical Care Center, Sabadell Hospital, Parc Taulí, 1.08028, Autonomous University of Barcelona, CIBER Enfermedades Respiratorias, Sabadell, Spain

## Abstract

Severe sepsis results in high morbidity and mortality. Immunomodulation strategies could be an adjunctive therapy to treat sepsis. Endotoxin is a component of gram-negative bacteria and plays an important role in the pathogenesis of septic shock when it is recognized by immune cells. Removal of endotoxin could be an effective adjunctive approach to the management of sepsis. Devices to adsorb endotoxin or inflammatory cytokines have been designed as a strategy to treat severe sepsis, especially sepsis caused by gram-negative bacteria. Polymyxin B-immobilized cartridge has been successfully used to treat patients with sepsis of abdominal origin. Although this cartridge was conceived to adsorb endotoxin, several other immunological mechanisms have been elucidated, and this device has also yielded promising results in patients with nonseptic respiratory failure. In this paper, we summarize the immune modulation actions of Polymyxin B-immobilized cartridge to explore its potential usefulness beyond endotoxin elimination.

## 1. Introduction

Sepsis is a major healthcare problem. Despite advances in supportive care of critically ill patients, sepsis remains an important cause of death worldwide in adults and children [1–3]. The Surviving Sepsis Campaign (SSC), which standardized the approach to sepsis, was recently updated [4]. Several efforts have been made to improve adherence to SSC guidelines [5, 6]. Nevertheless, mortality and costs are still high [2, 7, 8]. Sepsis is characterized by a complex systemic inflammatory response to a microbial pathogen. First, the presence of microorganisms in the bloodstream causes an innate immune response characterized by the stimulation of monocytes and release of proinflammatory cytokines and the activation of a medley of different immune pathways. Toll-like receptors (TLRs) play a key role in this initial immune activation, acting as innate immune system sensors through the recognition of highly conserved components of a variety of microorganisms [9]. The activation of TLRs induces an inflammatory response to control the infection, which results in local vasodilatation, release of various cytotoxic chemicals, and, hopefully, destruction of the invading pathogen. Many of these same components of inflammation that are beneficial in host defenses against infection can, under some circumstances, be deleterious, causing cell and tissue damage and hence multiple organ failure. Endotoxin, also known as lipopolysaccharide, is a component of gram-negative bacteria and a strong activator of TLR4. The recognition of endotoxin by immune cells is important in the pathogenesis of septic shock [10, 11].

Conventional therapy such as antibiotics and surgical procedures to remove the source of infection is crucial for treating sepsis, but these approaches cannot reverse the effects of the bacterial toxins already released into blood or of the endogenous mediators produced by the host in response to bacteria. Over recent years, numerous attempts have aimed to intervene in the inflammatory cascade. Attempts to stop the inflammatory cascade using antiendotoxin strategies such as monoclonal antibodies or vaccines have failed [12, 13]. Phospholipid emulsion to neutralize endotoxin does not improve outcome in septic patients [14]. A recent phase 2 trial found a nonsignificant trend toward better survival in patients with severe sepsis treated with eritoran tetrasodium, a TLR-4 antagonist [15].

Blood purification techniques including hemoperfusion, plasma exchange, and hemofiltration with hemoperfusion are associated with lower mortality in patients with sepsis as it has been demonstrated in a recent meta-analysis [16]. Removing endotoxin would be an effective adjunctive approach in the management of severe sepsis. Devices to remove endotoxin or inflammatory cytokines have been designed as a strategy to reduce the morbidity and mortality associated with sepsis, especially with sepsis due to gram-negative bacteria. These devices have also been successfully used in patients with sepsis due to gram-positive microorganisms and in patients with acute respiratory distress syndrome (ARDS), suggesting that they could have an immunomodulating action in addition to endotoxin elimination [17–21]. Indeed, many studies report additional beneficial immunological mechanisms for endotoxin removal devices. This review aims to summarize the immune modulation actions of Polymyxin B-immobilized cartridge to understand the potential usefulness of this device beyond endotoxin elimination.

## 2. Devices to Remove Endotoxin and Inflammatory Molecules

Over recent years, devices to eliminate endotoxin, inflammatory molecules such as cytokines and immune cells, have been designed to mitigate the deleterious effects of the inflammatory cascade. These extracorporeal devices act through convection or adsorption. A double lumen catheter for extracorporeal use is needed. Most of these devices are designed to combine the effect of molecules removal with renal replacement therapies (hemofiltration, dialysis, or hemodiafiltration). The biocompatibility of these devices is the main limitation for its use, thrombocytopenia and bleeding risk are the potential side effects [22].  Table 1 summarizes the mechanism of action and molecules removed by each membrane.

### 2.1. Polymyxin B-Immobilized Cartridge (Toraymyxin 20-R, Toray Industries, Japan)

Polymyxin B is a cationic polypeptide antibiotic with activity against gram-negative bacteria and a high affinity to endotoxin, but its intravenous use has been limited due to nephrotoxicity and neurotoxicity [23]. Since 1994, Polymyxin B has been fixed and immobilized with polystyrene fiber in a hemoperfusion column Polymyxin B-immobilized cartridge (PMX) that allows endotoxin removal without the toxic effects of this antibiotic. This treatment has been widely used in Japan for septic shock due to gram-negative bacteria, and its use was authorized in Europe in 1998. Recent studies support the safety and efficacy of this treatment [16, 24–26]. 

### 2.2. LPS Adsorber (Alteco Medical AB, Sweden)

This medical device designed for extracorporeal use contains a series of porous polyethylene plates coated with a peptide specific to endotoxin and has a high adsorption capacity. It has been used in patients with septic shock [27–29]. Yaroustovsky et al. [28] compared LPS adsorber and PMX hemoperfusion in a small sample of patients with gram-negative sepsis. The authors did not find differences in outcome. However, due to limitations of the study, the authors concluded that further studies were necessary to clarify the efficacy of LPS adsorber.

### 2.3. OXiris (Gambro-Hospal, France)

This AN-69 (polysulfone and polyacrylonitrile) based membrane adsorbs a large spectrum of plasma inflammatory mediators such as endotoxin and cytokines [30, 31]. To date, clinical experience with this device is limited, but two trials are underway in septic patients [32]. The results of these two trials are crucial to determine its usefulness compared with the current standard of care.

### 2.4. MATISSE-Fresenius System (Fresenius SE, Germany)

Based on the endotoxin-binding abilities of human albumin, this adsorber contains human serum albumin immobilized on polymethacrylate beads. Although in vitro experiments were promising, phase 2 study results have been disappointing [33].

### 2.5. Coupled Plasma Filtration Adsorption, CPFA Bellco, Italy

This extracorporeal treatment is based on nonspecific adsorption of cytokines and other proinflammatory mediators onto a specially designed resin cartridge, coupled with hemofiltration. This device does not adsorb endotoxin. Some studies have shown interesting results regarding hemodynamics and respiratory parameters [34–36]. This is a promising therapy, although further studies are necessary to determine its usefulness in septic patients. One clinical trial, COMPACT 2, is underway to clarify whether adding high doses of CPFA to current clinical practice can reduce hospital mortality in septic shock patients (ClinicalTrials.gov number NCT01639664) [37].

### 2.6. CytoSorb (Cytosorbents Inc., USA)

This extracorporeal device removes cytokines through adsorption to a high-surface-area biocompatible porous polymer sorbent. This device does not target endotoxin, but it does rapidly eliminate several key cytokines by adsorption in both in vitro and in vivo experiments [38, 39]. This device is very promising, but more studies in septic patients are needed.

Due to the broad information existing about safety and efficacy of Polymyxin B-immobilized cartridge, we will review the immunological mechanisms described in this treatment.

## 3. Immunological Mechanisms Described for Polymyxin B-Immobilized Cartridge

Polymyxin B-immobilized cartridges (PMX) are designed to bind endotoxin. However, other mechanisms of immunomodulation have also been described. Whereas some of these mechanisms are derived from endotoxin elimination, others result from direct action on other inflammatory molecules and cells or from a combination of endotoxin elimination and direct action on these mediators.  Table 2 summarizes these mechanisms.

### 3.1. Endotoxin Removal

**Figure 1:**
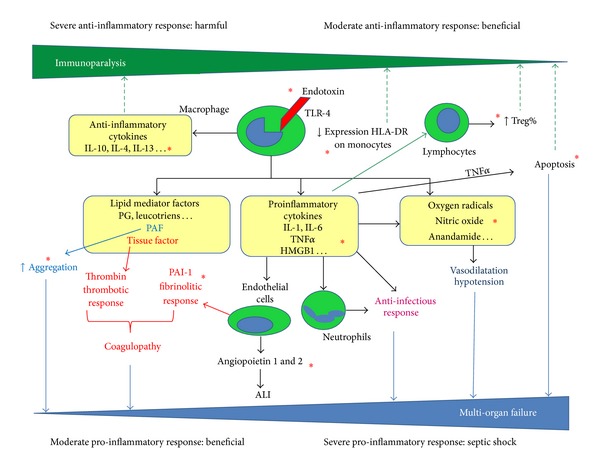
Proinflammatory and anti-inflammatory responses to endotoxin. Red asterisk: immunological mechanisms described for Polymyxin-B hemoperfusion in this review; TLR: toll-like receptor; IL: interleukin; T_reg_: T regulatory lymphocytes; TNF*α*: tumor necrosis factor alpha; HMGB1: high mobility group box protein 1; PAF: platelet activator factors; PAI: plasminogen activator inhibitor; ALI: acute lung injury.

Endotoxin is a major component of the outer membrane of gram-negative microorganisms [40]. Immune cells recognize endotoxin and other bacterial compounds through the TLR, a group of transmembrane proteins that play crucial roles in the host defense against invading pathogens [41]. During a gram-negative infection, TLR-4 recognizes endotoxin and originates a systemic inflammatory response in sepsis with potentially fatal effects in hosts. As a consequence, proinflammatory molecules such as interleukin-1 (IL-1) and tumor necrosis factor alpha (TNF*α*) are released and generate other cell responses in the inflammatory cascade (Figure 1). This increase in cytokines is followed by a major expression of tissue factor, which activates coagulation, and by an increase in nitric oxide synthesis, which induces vasodilation [42]. Endotoxin levels are high in septic patients [43, 44], but they are also high in critical patients without sepsis, such as patients undergoing cardiopulmonary bypass and those with chronic heart failure, chronic kidney disease, and other medical conditions [44–47]. In critical patients without gram-negative infection, elevated endotoxin levels are related to translocation of gut bacterial antigens and endotoxin into the bloodstream due to gut barrier dysfunction [48–50].

Polymyxin B binds endotoxin through hydrophobic and ionic interactions. Polymyxin B's hydrophobic amino acids (Phe, Leu) form hydrophobic bonds with lipid A fatty acid in endotoxin, and the amino groups of Polymyxin B form ionic bonds with the negatively charged phosphate groups of lipid A [51]. This binding results in an antibiotic-endotoxin complex that is highly effective in neutralizing the deleterious effects of endotoxin.

Many studies have reported diverse benefits of PMX hemoperfusion in septic patients, including improved hemodynamics [24, 25, 52–60], increased ratio of partial pressure arterial oxygen and fraction of inspired oxygen (PaO_2_/FiO_2_) [24, 54, 55, 57, 58, 60, 61], decreased 28-day mortality [24, 52, 53, 59], and decreased endotoxin levels [52, 53, 55, 58, 59, 62–64]. In a multicenter study, Vincent et al. [25] found that PMX hemoperfusion was safe and improved cardiac and renal function due to sepsis or septic shock; however, they could not demonstrate a reduction in mortality or in endotoxin levels from baseline to the end of treatment. In a systematic review of 28 studies, Cruz et al. [65] concluded that PMX hemoperfusion was associated with lower mortality (RR 0.53, 95% CI: 0.43–0.65) and improvements in mean arterial pressure (MAP), use of inotropes, and PaO_2_/FiO_2_. In 17 of these studies in which endotoxin levels were measured, endotoxin levels decreased by 33% to 80% after PMX hemoperfusion [65]. More recently, the same authors published the EUPHAS study [24], a prospective multicenter randomized controlled trial that enrolled 64 patients with severe sepsis or septic shock who underwent emergency surgery for intra-abdominal infection. Patients were randomized to conventional therapy or to conventional therapy plus two sessions of PMX hemoperfusion. After the results of the scheduled interim, analysis revealed that PMX hemoperfusion significantly improved hemodynamics and organ dysfunction and reduced 28-day mortality; the study was discontinued because it was considered unethical to deprive high risk patients of a potentially beneficial therapy; however, early discontinuation resulted in a modest sample size. This study did not measure endotoxin. Two adequately powered prospective trials are underway, and the results of these trials should elucidate the benefit of endotoxin removal (ClinicalTrials.gov numbers NCT01046669 and NCT01222663) [66, 67].

### 3.2. Elimination of Cytokines and Inflammatory Molecules

Several studies report a reduction in cytokines and inflammatory molecules in patients' plasma after PMX hemoperfusion [54, 56, 61, 68–70]. 

#### 3.2.1. Cytokines and Inflammatory Proteins

In patients with severe sepsis, Tani et al. [54] found reductions in endotoxin, TNF*α*, IL-6, IL-10, and plasminogen activator inhibitor-1 (PAI-1) activities after PMX hemoperfusion. In patients with ARDS, Kushi et al. [61] found a reduction in blood levels of PAI-1, neutrophil elastase (NE), and IL-8 after PMX hemoperfusion. NE is a protease that hydrolyzes lung elastin. In these patients, PaO_2_/FiO_2_ increased significantly after the treatment, and the authors related this increase to the elimination of IL-8 and NE. In another study, the same group reported a decrease in NE in 20 septic patients treated with PMX hemoperfusion [71]. In 12 patients with septic shock receiving conventional treatment plus two sessions of PMX hemoperfusion, Zagli et al. [72] found a decrease in IL6, IL10, and TNF*α* in patients' serum after the treatment, especially in survivors. Most authors attribute the decrease in cytokines and inflammatory molecules to the removal of endotoxin and to the effect of this removal on the inflammatory cascade. 

#### 3.2.2. High Mobility Group Box-1 Protein 

Patients with sepsis have increased high mobility group box-1 protein (HMGB1), a cytokine secreted by immune cells that triggers inflammatory mediators [73]. The receptor for advanced glycation end-products (RAGE) is involved in HMGB1 signaling. The inhibition of the HMGB-1-RAGE axis could be an effective therapeutic strategy for septic shock. Nakamura et al. [63] compared IL-6, HMGB1, and RAGE in serum between 15 patients with septic shock treated with PMX hemoperfusion and healthy volunteers. The levels of the three molecules decreased after PMX hemoperfusion and correlated with a decrease in endotoxin. Abe et al. [74] studied the effects of PMX hemoperfusion on HMGB1 in patients with acute exacerbation of idiopathic pulmonary fibrosis. PMX hemoperfusion both significantly decreased the serum HMGB-1 level and improved the PaO_2_/FiO_2_ ratio. Moreover, HMGB-1 was detected in washing medium from the PMX column, suggesting that the decrease in this molecule was not only secondary to endotoxin removal but also to direct removal by the device. A recent study exploring the meaning of HMGB-1 levels in 60 patients with septic shock treated with PMX hemoperfusion found a significant positive correlation between the Sequential Organ Failure Assessment (SOFA) score and HMGB-1 level (*P* < 0.05). The authors concluded that HMGB-1 is a useful prognostic biomarker in sepsis-induced organ failure in patients undergoing PMX hemoperfusion, but formal establishment of the utility of HMGB-1 as a prognostic biomarker still remains to be performed [68].

#### 3.2.3. Vascular and Coagulation Proteins

PAI-1, a marker of vascular endothelial cell activation elevated by endotoxin and cytokines, is one of the fibrinolysis inhibitory factors. PAI-1 levels decrease after PMX hemoperfusion, decreasing the stimulation of vascular endothelial cells [54, 55, 61]. PMX hemoperfusion may have a role in modulating fibrinolysis and inhibiting the development of ischemic organ dysfunction in sepsis.

Angiopoietin-1 is a positive regulator of blood vessel development, remodeling, and maturation. Angiopoietin-2 is a competitive inhibitor of angiopoietin-1. Angiopoietin-1 and -2 play a contributory role in the pathogenesis of acute lung injury (ALI) in septic patients. Angiopoietin-1 reduces pulmonary inflammation and permeability. Angiopoietin-2 interferes with angiopoietin-1, resulting in pulmonary inflammation and increased permeability. Ebihara et al. [75] reported that PMX hemoperfusion could ameliorate the angiopoietin balance in septic patients with ALI.

Vascular endothelial growth factor (VEGF) is a pluripotent growth and permeability factor that has a broad impact on endothelial cell function. VEGF also plays a role in several acute and chronic lung diseases [76]. Oishi et al. [69] recently studied nine patients with acute exacerbation of idiopathic pulmonary fibrosis treated with conventional therapy and PMX hemoperfusion 6 hours/day on two successive days. They found a high concentration of cytokines and VEGF in the eluate from used PMX cartridge fibers, and the clinical improvement in these patients correlated with the amount of VEGF in the eluate. This is the first study to demonstrate that cytokines and VEGF can be directly adsorbed by PMX hemoperfusion independently from endotoxin removal. The authors suggest that cytokines can bind to PMX hemoperfusion fibers directly through ionic/hydrophobic interactions like endotoxin or indirectly via heparin coated in the fibers. 

#### 3.2.4. Other Molecules

Anandamide is an intrinsic cannabinoid that has been related with hypotension in septic shock, although currently its direct link to sepsis is only established in a small patient population. Polymyxin-B directly binds anandamide in vitro [77]. One study in 24 patients with septic shock treated with PMX hemoperfusion found that anandamide levels decreased after PMX hemoperfusion in the nine patients who survived; the authors conclude that removal of anandamide by PMX hemoperfusion, whether directly or as a result of endotoxin elimination, could be key to successful septic shock treatment [78]. Further studies are necessary to elucidate the effect of PMX hemoperfusion on anandamide, in order to establish it as a useful treatment for hypotension. 

Elevation of nitric oxide (NO) plays an important role in septic patients, producing vasodilatation and hypotension. Nakamura et al. [79] compared NO breakdown products in urine in 20 patients with PMX hemoperfusion, 15 patients with conventional therapy, and 20 healthy controls. They found that septic patients increased NO production and that PMX hemoperfusion reduced NO levels and thus increased blood pressure. 

Troponin is a biomarker that may be elevated in septic patients as a result of subclinical myocardial cell damage. Nakamura et al. [80] found increased troponin T in septic patients compared to nonseptic patients and age-matched healthy controls; interestingly, troponin T decreased after PMX hemoperfusion *P* < 0.05.

Erythropoietin levels may be higher in patients with sepsis; erythropoietin levels decrease after PMX hemoperfusion and could be a prognostic indicator in patients with septic shock [70]. 

### 3.3. Removal of Cells and Phenotype Change

During sepsis, different populations of leukocytes are activated and change their adhesive phenotype. The capture of leukocytes through extracorporeal blood purification could alter the immune response to sepsis [81]. After ex vivo perfusion of heparinized blood from patients with sepsis and septic shock through PMX hemoperfusion in a laboratory circuit, Kumagai et al. [82] found significant decreases in neutrophils (78%), monocytes (70%), and lymphocytes (10%). This marked reduction in white blood cells should be attributed mostly to the reduction in the circulation of proinflammatory cytokines that induce cell activation and proliferation, as opposed to a direct effect of removal of these cells by the cartridge. Nishibori et al. [83] examined the PMX hemoperfusion filters after treating 4 patients with sepsis; PMX hemoperfusion bound monocytes from the peripheral blood leucocytes. PMX hemoperfusion could produce a beneficial effect by reducing the interaction between monocytes and functionally associated cells, including endothelial cells.

The inflammatory response in sepsis involves activation of platelets. High levels of platelet activator factor (PAF) have been observed in sepsis. Nakamura et al. [52] studied the effect of PMX hemoperfusion on platelet activation, comparing 30 patients treated with conventional therapy plus PMX hemoperfusion and 20 patients with conventional therapy alone. Survival was 60% in the group that received PMX hemoperfusion and 30% in the group that received only conventional treatment. Septic patients had increased PAF (P-selectin, platelet factor 4, and *β*-thromboglobulin), and PMX hemoperfusion reduced the levels of PAF.

### 3.4. Effect on Immunoparalysis

The human body undergoes a biphasic immunological reaction in sepsis. A proinflammatory reaction takes place, marked by the release of proinflammatory cytokines like TNF*α*, as a reaction to the bacterial toxins. On the other hand, a counter regulatory anti-inflammatory reaction arises. This phase acts as negative feedback on the inflammation by inhibiting the proinflammatory cytokines. The persistence of a marked compensatory anti-inflammatory response is called “immunoparalysis” (Figure 1). This pronounced immunosuppressive state adversely affects immune function, making the patient vulnerable to opportunistic infections [84]. These two phases of sepsis may occur simultaneously with a lasting anti-inflammatory response in later phases [85]. Most septic patients survive the initial proinflammatory phase, but they die during this second stage. Strategies to stimulate this immunoparalysis phase of sepsis as IFN-*γ* and granulocyte-macrophage colony-stimulating factor (GM-CSF) have been developed in animals, but extensive clinical studies are needed to test their safety and efficacy [86].

Recently, Ono et al. [56] showed that the expression of the surface antigens, HLA-DR on monocytes and CD16 on granulocytes, is extremely decreased in patients with septic shock and that PMX hemoperfusion beneficially increases this expression on these leukocytes. Thus, PMX hemoperfusion might help septic patients recover from immunoparalysis. 

The regulatory T cells (T_reg_) that express CD4, CD25, and Foxp3 comprise a small percentage of the T-lymphocyte population in the immune system, but they are central to the maintenance of immunological homeostasis and tolerance. In septic patients, the percentage of T_reg_ is increased, and this presumably contributes to sepsis-induced immunosuppression. Polymyxin-B induces T_reg_ cell death in mice through the modulation of the purinergic P2X7 receptor [87]. Ono et al. [88] studied the effect of PMX hemoperfusion on the recovery from the immunosuppression owing to septic shock. T_reg_, IL-6, and IL-10 were higher in patients with septic shock than in patients with sepsis. After PMX hemoperfusion, T_reg_ cells, Il-6, and IL-10 decreased. In survivors, the decrease in T_reg_ cells was accompanied by an increase in CD4+ cells. Although further studies are necessary to confirm a causative relationship between T_reg_ depletion and PMX hemoperfusion in septic patients, this mechanism could explain why PMX hemoperfusion can be useful in patients without endotoxemia or in those with gram-positive sepsis [89, 90]. The authors also suggest that the second PMX hemoperfusion treatment might provide additional benefits for recovery from immunoparalysis. This study sheds new light on the benefits of treating septic patients with PMX hemoperfusion beyond endotoxin removal.

Apoptosis, programmed cell death, is an energy-dependent process [91]. Endotoxin may cause an inappropriate activation of proapoptotic pathways in immune cells during sepsis, and this may contribute to the impaired immune response that characterizes sepsis [92]. Endotoxin can also cause apoptosis of renal tubular cells through Fas-mediated and caspase-mediated pathways [93]. Cantaluppi et al. [94] tested the hypothesis that PMX hemoperfusion might prevent gram-negative sepsis-induced acute renal failure by reducing the activity of proapoptotic circulating factors. They randomized 16 patients with gram-negative sepsis to receive standard care or standard care plus PMX hemoperfusion. Proapoptotic activity was significantly reduced in the plasma of the PMX hemoperfusion group, with decreases in Fas upregulation and caspase activity, and these patients also had improved renal function.

## 4. Usefulness in Acute Respiratory Failure

Several studies have found that PMX hemoperfusion has beneficial effects on oxygenation in patients with sepsis [24, 61, 65]. Moreover, PMX hemoperfusion has been successful in patients with influenza A infection [89, 95], ARDS in drug-induced injury [96, 97], interstitial pneumonia [19, 20, 98], and idiopathic fibrosis [18, 69, 74, 99]. Mechanisms other than endotoxin removal could explain the beneficial effects of PMX hemoperfusion in patients with respiratory failure. 

Chemical mediators have an important role in the pathogenesis of ARDS, and decreasing them through direct or indirect removal could be beneficial in ARDS patients. Kushi et al. [61] found decreases in PAI1, neutrophil elastase (NE), and IL-8 in ARDS after PMX hemoperfusion. Abe et al. [74] studied the role of decreases in HMGB1 after PMX hemoperfusion in patients with acute exacerbation of idiopathic fibrosis. When the same authors investigated the effects of PMX hemoperfusion in a retrospective multicenter study of 160 patients with acute exacerbation of idiopathic pulmonary fibrosis or interstitial pneumonia, they found that the PaO_2_/FiO_2_ ratio significantly increased after PMX hemoperfusion [18]. They concluded that PMX hemoperfusion might be an effective adjunctive therapy for these patients, although the mechanisms underlying the benefits of the treatment are uncertain. Likewise, Hara et al. [20] reported that PMX hemoperfusion resulted in improved PaO_2_/FiO_2_ ratio 72 hours and 1 week after treatment in 33 patients with acute exacerbation of interstitial pneumonia. Tsushima et al. [100] treated 20 patients with ARDS with PMX hemoperfusion and compared the outcomes with a historical control group. They found improved PaO_2_/FiO_2_ ratio and survival; however, the methodology of the study limits its power to draw conclusions.

Collectively, the matrix metalloproteinases (MMP) are capable of degrading all kinds of extracellular matrix proteins. MMP-9 is a protease involved in the degradation of the basement membrane, a part of the extracellular matrix associated with the alveolar epithelium and vascular endothelium. MMP-9 is essential for the remodeling of basement membranes in various inflammatory lung diseases, including ARDS. Increased amounts of MMP-9 in the vasculature are likely to enhance vascular permeability and to facilitate cell homing and inflammatory remodeling. Nakamura et al. [62] studied the effect of PMX hemoperfusion on MMP levels in ARDS patients by treating 12 ARDS patients with two sessions of PMX hemoperfusion and comparing their laboratory data with those of healthy controls. After treatment, the PaO_2_/FiO_2_ ratio and hemodynamic parameters clearly improved. Patients with ARDS had significantly higher levels of MMP-9 than controls. After PMX hemoperfusion, MMP-9 decreased and chest X-ray findings improved. However, the precise mechanism is still unclear. The authors suggest the need for more studies to elucidate the beneficial effect of PMX hemoperfusion in ARDS. In a pilot study of 16 patients, Abe et al. [101] studied the effects of PMX hemoperfusion for acute exacerbation of interstitial pneumonia and demonstrated neutrophil adsorption and a decrease in MMP-9.

In recent years, the mediators S100A12 and RAGE have drawn attention as specific markers of ALI [102]. The expression of S100A12 in neutrophils increases in the presence of endotoxin. Takahashi et al. [103] studied the changes in serum S100A12 and RAGE after PMX hemoperfusion in postoperative septic shock. They found a significant decrease in S100A12 in serum after PMX hemoperfusion and an improvement in PaO_2_/FiO_2_ ratio but no decrease in RAGE. The authors attributed the decrease in S100A12 to the concomitant decrease in endotoxin.

As mentioned above, Oishi et al. [69] found cytokines and VEGF in the eluate from PMX hemoperfusion cartridges used to treat patients with acute exacerbation of pulmonary fibrosis, suggesting a new explanation for the improvement in oxygenation in nonseptic patients treated with PMX hemoperfusion.

## 5. Conclusions

In recent years, many studies have shown that PMX hemoperfusion is a promising strategy for immunomodulation in septic shock, and two ongoing clinical trials will be key in determining its usefulness. 

Although most studies have focused on the removal of endotoxin as the principal mechanism through which PMX hemoperfusion improves outcome in sepsis, other studies have revealed mechanisms involving diverse immunological pathways through which PMX hemoperfusion could improve outcome not only in sepsis but also in non-septic respiratory failure. However, these studies are limited by their small samples, their observational and in some cases retrospective design, and the lack of control groups in many cases. Well-designed clinical investigations with larger samples are needed to confirm these findings.

It is interesting to note that the elimination of endotoxin brings about a reduction in many inflammatory molecules and cells involved in the inflammatory cascade. Endotoxin removal devices act at the onset of this complex cascade, and their benefits in terms of immunomodulation are encouraging. Only a part of the consequences of endotoxin elimination has been studied and summarized in this review. Future studies might reveal other mediators and cells involved in sepsis that might be altered after endotoxin removal. 

Some of the studies reviewed here found mediators and cells in the eluate from PMX hemoperfusion cartridges or by direct examination of the filter. Further studies are necessary to elucidate how PMX hemoperfusion eliminates these molecules, whether through ionic/hydrophobic interactions like in endotoxin removal or indirectly via heparin that coats the fibers.

Additional mechanisms could potentially explain why PMX hemoperfusion can be beneficial in gram-positive sepsis or non-septic respiratory failure. Endotoxin can be elevated in other medical conditions apart from gram-negative infections, and its removal could also partially explain this benefit. The immunomodulating effects of PMX hemoperfusion in patients with interstitial pneumonia and acute exacerbations of pulmonary fibrosis are especially interesting, given the high mortality associated with these conditions. However, well-designed clinical trials are needed to assess the efficacy of PMX hemoperfusion in these medical conditions. 

Future potential directions such as combination of different hemoperfusion devices to treat septic patients, in order to alter the host inflammatory response in more than one step, are currently speculative. Technically, it could be viable, but no experience has been reported to date.

In summary, antimicrobial therapy, surgical treatment of the focus of infection, and hemodynamic stabilization are crucial in the treatment of severe sepsis. PMX hemoperfusion is an effective adjunctive treatment in septic shock. It seems that PMX hemoperfusion might have other beneficial immunological mechanisms in addition to endotoxin removal; however, the limited evidence suggests that we must be cautious with other indications for PMX hemoperfusion, and future studies are necessary.

## Figures and Tables

**Table 1:** Devices designed to remove endotoxin and cytokines in patients with septic shock.

Device	Company	Composition	Mechanism	Substance eliminated
Toraymyxin 20R	Toray Industries, Japan	Polymyxin B covalently bound to polypropylene-polystyrene fibers fabric	Adsorption	Endotoxin

LPS adsorber	Alteco Medical, Sweden	Synthetic polypeptide bound to porous polyethylene discs	Adsorption	Endotoxin

oXiris	Gambro-Hospal, France	AN69-based membrane, surface treated with a polyethyleneimine (PEI) and grafted with heparin	Adsorption Convection	Endotoxin Cytokines

MATISSE	Fresenius SE, Germany	Human serum albumin immobilised on polymethacrylate beads	Adsorption	Endotoxin

CPFA	Bellco, Italy	Polyethersulfone Plasma filter with adsorption on an unselective hydrophobicresin cartridge, and a synthetic high-permeability polyethersulfone hemofilter for continuous hemofiltration	Adsorption Plasma filtration	Cytokines

Cytosorb	Cytosorbents, USA	Polystyrenedivinyl benzene copolymer beads with a biocompatible polyvinylpyrrolidone coating.	Adsorption Convection	Cytokines

**Table 2:** Summary of mechanisms described for polymyxin B-immobilized cartridge hemoperfusion.

Molecules	Description	Effect of PMX	Clinical features after PMX	References
Endotoxin	Component of the external membrane of gram-negative microorganisms, recognized by immune cells	↓	Interruption of inflammatory cascade	[52–55, 58, 59, 62–64]

IL-1; IL-6; IL-8; IL-9; IL-10; IL-12; IL-17; *α*TNF	Pro- and anti-inflammatory cytokines; their overproduction is deleterious in sepsis	↓	Decrease in the excessive systemic host inflammatory response to infection	[54, 61, 63, 68–70, 72, 88]

Plasminogen activator inhibitor (PAI-1)	Component of the coagulation system that downregulates fibrinolysis in the circulation, favoring coagulation	↓	Regulation of fibrinolysis and reversal of the occurrence of sepsis-associated thrombosis	[54, 55, 61]

Neutrophil elastase	Protease that hydrolyzes lung elastase and other proteins	↓	Reduction of pulmonary tissue destruction	[61, 71]

High mobility group box protein 1, HMGB-1; receptor for advanced glycation end-products (RAGE), S100A12	HMBG-1 is a cytokine to trigger inflammatory mediators; RAGE is a receptor involved in HMBG-1 signaling; S100A12 is a mediator involved in acute lung injury	↓	Decrease in the excessive systemic host inflammatory response to infection	[63, 74, 102]

Anandamide	Intrinsic cannabinoid that induces hypotension in septic shock	↓	Decrease in septic shock-associated hypotension	[78]

Nitric oxide	Produces vasodilatation and hypotension	↓	Decrease in septic shock-associated hypotension	[79]

Erythropoietin	Protein that controls red blood cells production, elevated in sepsis	↓	Prognostic biomarker in sepsis	[70]

Troponin T	Protein found in cardiac muscle	↓	Decrease in myocardial cell damage	[80]

Angiopoietin-1 and -2	Angiopoietin-1 reduces pulmonary inflammation and permeability. Angiopoietin-2 interferes with angiopoietin-1, resulting in pulmonary inflammation and increased permeability	Balance	Decrease in acute lung injury	[75]

Vascular endothelial growth factor (VEGF)	Growth factor involved in several acute and chronic lung diseases	↓	Improvement of lung function	[69]

Monocytes, neutrophils, and lymphocytes	Immune cells involved in inflammatory response	↓	Decrease in the interaction between monocytes and functionally associated cells, decreasing inflammatory response, and decrease in neutrophil and lymphocyte response	[82, 83]

Platelet activator factors (PAF) (P-selectin, *β*-Thromboglobulin, Platelet factor 4)	PAF stimulates platelets, increasing procoagulation status in sepsis	↓	Decrease inprothrombotic status	[52]

HLA-DR and CD16 expression monocytes on granulocytes	Surface antigen expressions HLA-DR and CD-16 are decreased in sepsis	↑	Increasing in surface antigen expression on immune cells helps the recovery from immunoparalysis in sepsis	[56]

CD4+CD25+Foxp3+ Treg	T-lymphocytes, responsible formaintaining immunological homeostasis and tolerance, are increased in sepsis	↓	Recovery from immunoparalysis in sepsis	[88]

Apoptotic factors (Fas- and caspase-mediated)	Factors that activate cell programmed death of tubular cells	↓	Improvement in renal function by reduction of proapoptotic factors	[94]

Metalloproteinase MMP9	Protease involved in degradation of the basement membrane associated with the alveolar epithelium	↓	Decrease in alveolar destruction and improvement in respiratory function	[62, 101]

IL: interleukin; PMX: polymyxin-B immobilized cartridge.
